# Bacterial Culture of Tear Duct Infections Secondary to Congenital Nasolacrimal Duct Obstructions

**DOI:** 10.1155/2022/9954634

**Published:** 2022-03-28

**Authors:** Weiming Yang, Li Shen, Anken Wang, Meiyan Li, Chenhao Yang

**Affiliations:** ^1^Department of Ophthalmology, Children's Hospital of Fudan University, Shanghai, China; ^2^Department of Ophthalmology and Optometry, Eye and ENT Hospital, Fudan University, Shanghai, China NHC Key Laboratory of Myopia (Fudan University), Shanghai Research Center of Ophthalmology and Optometry

## Abstract

**Purpose:**

To investigate the microbial profile of congenital nasolacrimal duct obstruction (CNLDO) in Chinese children.

**Methods:**

We retrospectively reviewed the medical records of 330 consecutive children (330 eyes) who were diagnosed with tear duct infections secondary to CNLDO and were admitted to the Children's Hospital of Fudan University from January 2013 to January 2020. Bacterial cultures were grown from tear duct samples of each patient. Samples from conjunctival secretions were cultivated on blood or chocolate agar. Clinically significant bacterial growth was reported.

**Results:**

Of the 330 eyes considered, 62.7% (207/330) were associated with positive bacterial cultures. A total of 223 isolates were detected from 207 culture-positive eyes. Among the 223 isolates, 52.0% (116/223) were Gram-positive bacteria and 47.1% (105/223) were Gram-negative bacteria. The most prevalent Gram-positive bacteria were *Streptococcus viridans* (67 isolates, 30%), followed by *Staphylococcus aureus* (36 isolates, 16.1%) and *Streptococcus pneumoniae* (5 isolates, 2.2%). The most prevalent Gram-negative bacteria were *Neisseria* (nonpathogenic) (25 isolates, 11.2%), followed by *Escherichia coli* (16 isolates, 7.2%) and *Haemophilus influenzae* (16 eyes, 7.2%). Antibiotic susceptibility test results suggested that both Gram-positive and Gram-negative bacteria were highly sensitive to most of the tested antibiotics.

**Conclusions:**

*S. viridans* and *S. aureus* are the most prevalent bacteria in tear duct infections secondary to CNLDO. Broad-spectrum antibacterial eye drops are suggested as empirical antibiotic treatments.

## 1. Introduction

Congenital nasolacrimal duct obstruction (CNLDO) is a common disease in newborns and the most common cause of neonatal epiphora (excessive tearing) [1], [2]. CNLDO has been reported to occur in 1.75–6% of newborns [3], [4]. CNLDO often occurs when Hasner's membrane at the end of the nasolacrimal duct is not broken, which results in the blockage or retention of tear fluid in the lacrimal sac. Symptoms caused by CNLDO may occur shortly after birth [3–5]. CNLDO symptoms include lacrimation and pus formation, which can cause eczema blepharitis and eczematous dermatitis. Lacrimal sac secretions can affect the balance of conjunctival sac flora.

Bacterial culture of conjunctival sac secretions is a commonly used clinical method for identifying pathogenic bacteria, which guides the treatment of patients. To our knowledge, few studies have reported the bacterial etiology of CNLDO in children [4], [6–8], and no study has focused on bacterial etiology of CNLDO in Chinese children. Herein, in the present study, we retrospectively reviewed medical data of patients from Children's Hospital of Fudan University to describe the spectrum of microbial isolates and evaluate the antibiotic susceptibility of identified organisms in children with CNLDO.

## 2. Methods

### 2.1. Patients

A retrospective review of medical records of consecutive patients who were diagnosed with tear duct infections secondary to CNLDO at the Children's Hospital of Fudan University from January 2013 to January 2020 was conducted. The inclusion criteria were as follows: patients aged <4 years and not under any antibiotic treatment. The exclusion criteria were as follows: patients with external ocular diseases, patients who had undergone ocular surgery in the last 6 months, and patients with systematic diseases.

### 2.2. Ophthalmic Examination

CNLDO was diagnosed based on clinical features of mucopurulent discharge and epiphora. When the pressure was applied to the lacrimal sac, reflux of mucoid or mucopurulent material from the punctum was observed. All patients were administered empirical antibiotic treatments after culture samples were carefully collected. Treatment scenarios were then changed in accordance with the results of microbiological agent sensitivity tests.

### 2.3. Microbiological Analysis

To obtain a conjunctival sac culture, the lower conjunctival fornix was exposed by pulling on the lower lid with a finger and swabbing the area with a cotton stick. Samples were then inoculated in blood or chocolate agar media (Shanghai Yihua Biotechnology Co., Ltd). Blood agar cultures were incubated for 48–72 h at 37°C with 5% carbon dioxide; the presence of a single colony was considered a positive test result. Bacterial identification was performed using a Bruker mass spectrometer (matrix-assisted laser desorption/ionization time of flight mass spectrometry, MALDI-TOF). Antibiotic susceptibility testing was performed in accordance with bacterial culture results. Antimicrobial susceptibility tests were carried out using the Kirby–Bauer method or automated systems (Vitek2 Compact, France), with interpretation according to the Clinical and Laboratory Standards Institute (CLSI). Reference strains such as *Staphylococcus aureus* ATCC 25923, *Enterococcus faecalis* ATCC 29212, *Escherichia coli* ATCC25922, *Pseudomonas aeruginosa* ATCC27853, and *Streptococcus pneumoniae* ATCC49619 were included to ensure reproducibility of the antibiotic susceptibility testing procedure. In the present study, *Neisseria* species other than *Neisseria gonorrhoeae* and *Neisseria meningitidis* are considered nonpathogenic *Neisseria*.

### 2.4. Statistical Analysis

Data were analyzed using Stata 9.0 software (StataCorp, Texas, USA) and presented as a mean ± standard deviation (SD) or median value.

## 3. Results

A total of 330 patients (204 men and 126 women) with 330 affected eyes were included in the present study. The mean age of the patients at the time of examination was 2.2 ± 5.1 months and ranged from 0.1 to 48 months. Changes in the rate of positive test results per year are given in Table 1 and range from 45.83% to 70.73%.

Of the 330 eyes evaluated, 62.7% (207/330) were associated with positive bacterial cultures. Among the positive cases identified, 64.3% (133/207) were male and 35.7% (74/207) were female. In 92.3% (191/207) of the eyes that tested positive via bacterial culture, one bacterial species was identified, while more than one bacterium was identified in 7.7% (16/207) of positive eye cultures. The bacterial distribution is given in Table 2. A total of 223 isolates were detected in 207 positive eyes.

Among 223 isolates, 52.0% (116/223) were Gram-positive bacteria and 47.1% (105/223) were Gram-negative bacteria. Fungal isolates accounted for 0.9% (2/223) (*Candida albicans* and *Candida parapsilosis*) of positive cultures. The most frequently identified organism was *Streptococcus viridans* (67 isolates, 30.0%), followed by *Staphylococcus aureus* (36 isolates, 16.16%), *Neisseria* (nonpathogenic) (25 isolates, 11.2%), *Escherichia coli* (16 isolates, 7.2%), and *Haemophilus influenzae* (16 isolates, 7.2%) (Table 2).

**Figure 1:**
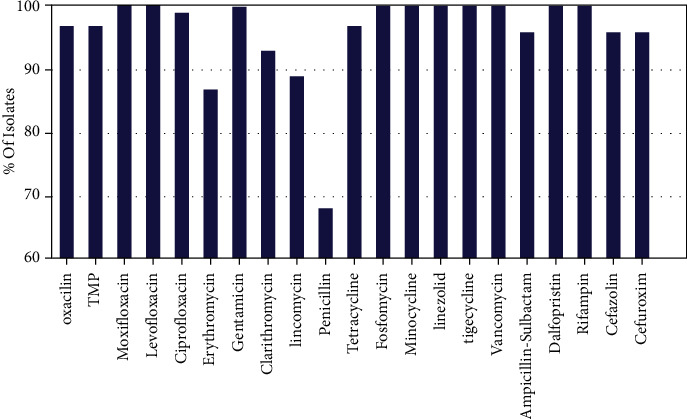
Antibiotic susceptibility of Gram-positive isolates.

**Figure 2:**
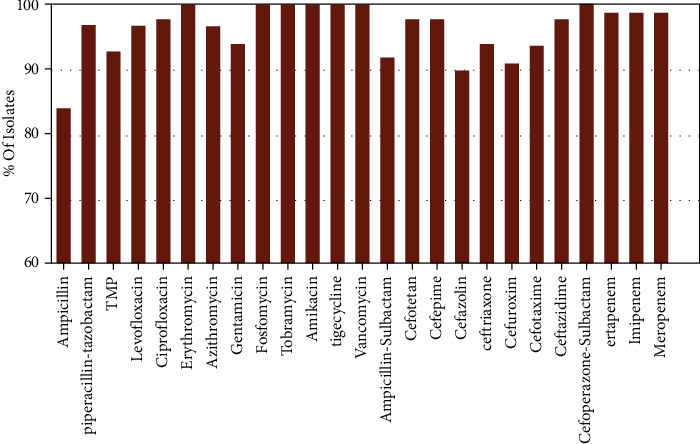
Antibiotic susceptibility of Gram-negative isolates.

The antibiotic susceptibility results are shown in Figures 1 and 2. Gram-positive bacteria were highly sensitive to moxifloxacin (100%, 116/116), levofloxacin (100%, 116/116), gentamicin (100%, 116/116), fosfomycin (100%, 116/116), minocycline (100%, 116/116), linezolid (100%, 116/116), tigecycline (100%, 116/116), vancomycin (100%, 116/116), dalfopristin (100%, 116/116), and rifampin (100%, 116/116). A high degree of resistance to penicillin was observed (68%, 79/116). Gram-negative bacteria also presented a high sensitivity to the tested antibiotics ranging from 83.81% to 100%.

## 4. Discussion

Treatment methods for CNLDO include topical antibiotic eye drops, lacrimal sac massage, lacrimal passage irrigation, and lacrimal passage probing. Broad-spectrum topical antibiotic eye drops are commonly used to treat discharge or pus associated with CNLDO. Topical antibiotic eye drops are very effective for decreasing mucopurulent discharge. Although serious complications secondary to CNLDO are rare [9], knowledge of the bacterial profile of CNLDO can facilitate the selection of effective antimicrobial agents, which may decrease microbial flora and purulent discharge, and prevent the development of serious infective complications. The bacterial spectra of CNLDO vary among different ethnic populations. The present study focused on the bacterial spectrum and associated antibiotic susceptibility of Chinese children.

In the present study, 52% percent of isolates were Gram-positive bacteria and 47.1% were Gram-negative bacteria. Many species of bacteria were detected. The most frequently identified organism was *S. viridis*, followed by *S. aureus*. In partial accordance with the results in our study, Usha et al. [4] reported a positive bacterial culture rate of 83% (197/238). Fifty-seven percent of isolates were Gram-positive bacteria, and the most frequently identified isolate was *S. pneumonia*. Gram-negative bacteria accounted for 43% (93/238) of identified bacteria, and *H. influenzae* was most frequently observed. Contrary to our results, Bekmez et al. [6] reported that positive bacterial proliferation test results were noted in 28.6% of patients, which is significantly lower than that in our study. Eighty percent (*n* = 16) of culture-positive bacteria were Gram-negative bacteria and 20% (*n* = 4) were Gram-positive bacteria. The most commonly observed bacteria were *H. haemolyticus* (20%) and *H. influenzae* (20%). Underlying reasons for discrepancies between studies may be study population ethnicity differences throughout different regions and environmental differences; therefore, studies that assess bacterial communities in specific populations are worthwhile and needed to fully understand the disease.

In the present study, most of the detected Gram-negative bacteria were conditional pathogens (*H. influenzae* and *E. coli*). The spectrum and incidence of pathogens differed across regions. In recent years, the number of conditional pathogens and Gram-negative bacilli has been increasing, and fungal infections have also been detected. In 1990, Bareja et al. [10] investigated the profile and incidence of dacryocystitis in an Indian infantile population. Gram-positive organisms accounted for 85.7% of isolates in their study, which is higher than that of our and other studies performed in Chinese populations [4], [6]. The underlying reason for this difference may be the widespread use of antibiotics, which results in etiological ophthalmic bacterial infection changes.

Several studies have reported that as high as 11.7–33% of cases of CNLDO contain two or more bacterial isolates [4], [11], [12]. Ghose and Mahajan V. M. [13] investigated the spectrum and incidence of fungi in congenital dacryocystitis. Fungi were detected in 30.2% of patients. Similarly, in the present study, 7.7% (16/207) of the eyes were found to contain two bacteria. In addition, 0.9% (2/223) were found to be fungal isolates (*C. albicans* and *C. parapsilosis*). The reason fungi are found on the ocular surface remains unclear. It was speculated that this may result from chronic fungal dacryocystitis, or secondary development of infection may occur in stagnant fluids of an obstructed lacrimal sac.

Antibiotic susceptibility testing is of great clinical significance in disease diagnosis and treatment, especially for treatment selection. In the present study, both Gram-positive and Gram-negative bacteria were highly sensitive to the majority of antibiotics considered. This may have been affected by fact that patients in the present study had no history of topical or systematic antibiotic use.

A limitation of this study is its retrospective design; therefore, future studies with a prospective design are warranted. The strength of this study is that only patients of 0–4 years old were included. In addition, the exclusion of patients that had received antibiotic treatment at the time of bacterial culture helped us better understand the bacterial spectrum of the population and assess susceptibility to infection in patients with CLNDO.

In conclusion, the most prevalent bacteria in tear duct infections secondary to CLNDO are *S. viridans*, followed by *S. aureus*, *Neisseria* (nonpathogenic), *E. coli*, and *H. influenzae*. Both Gram-positive and Gram-negative bacteria presented high sensitivity to most antibiotics assessed. These results could help decision-making in the clinic, especially in antibiotics selection.

## Figures and Tables

**Table 1:** The detection rate of bacteria in different years.

Year	Total no. of eyes	No. of positive eyes	Positive rate (%)
2013	44	30	68.18
2014	67	44	65.67
2015	41	21	51.21
2016	60	35	58.33
2017	51	35	68.62
2018	41	29	70.73
2019	24	11	45.83
2020 (Jan)	2	2	100
Total	330	207	—

**Table 2:** Bacteriology of congenital nasolacrimal duct obstruction.

Classification	Bacteria	No.	Percentage (%)
Gram-positive cocci	*Staphylococcus* species	42	18.83
	*Staphylococcus aureus*	36	16.14
	MRSA	6	2.69
	*Viridans streptococci*	67	30.04
	*Streptococcus pneumoniae*	5	2.24
	*Streptococcus constellatus*	1	0.44
	*Streptococcus agalactiae*	1	0.44

Gram-negative cocci	*Branhamella catarrhalis*	9	4.03
	^*∗*^ *Neisseria* (nonpathogenic)	25	11.21

Gram-negative rods	*Klebsiella ozaenae*	15	6.73
	*Citrobacter braakii*	2	0.89
	*Escherichia coli*	16	7.17
	*Stenotrophomonas maltophilia*	1	0.44
	*Haemophilus influenzae*	16	7.17
	*Serratia marcescens*	2	0.89
	*Haemophilus parainfluenzae*	3	1.35
	*Haemophilus haemolyticus*	11	4.93
	*Pseudomonas aeruginosa*	4	1.79
	*Enterobacter cloacae*	1	0.44
Total	—	223	100

MRSA, methicillin-resistant Staphylococcus aureus. ^*∗*^*Neisseria* species other than *Neisseria gonorrhoeae* and *Neisseria meningitidis* are considered nonpathogenic *Neisseria*.

## Data Availability

The datasets used and/or analyzed during the current study are available from the corresponding author upon request.
